# Development and Validation of Ultrahigh-Performance Liquid Chromatography Coupled with Triple Quadrupole Mass Spectrometry Method for Quantitative Determination of Ten Active Compounds in Ge-Gen-Jiao-Tai-Wan

**DOI:** 10.1155/2022/4713799

**Published:** 2022-04-10

**Authors:** Wenbo Wang, Shuangquan Zhu, Hao Chen, Ning Wu, Han Chen, Dongsheng Wang

**Affiliations:** ^1^Department of Integrated Traditional Chinese and Western Medicine, Xiangya Hospital, Central South University, Changsha 410008, China; ^2^National Clinical Research Center for Geriatric Diseases, Xiangya Hospital, Central South University, Changsha 410008, China; ^3^Department of Gynecology, The Second Affiliated Hospital of Hunan University of Chinese Medicine, Changsha 410005, China; ^4^Department of Clinical Laboratory, The First Affiliated Hospital of Hunan University of Chinese Medicine, Changsha 410007, China; ^5^Changsha Social Work College, Changsha 410116, China

## Abstract

A rapid, accurate, and sensitive method for the simultaneous determination of 10 main components, namely puerarin, daidzin, coptisine, epiberberine, jatrorrhizine, berberine, palmatine, coumarin, daidzein, and cinnamic acid in Ge-Gen-Jiao-Tai-Wan, was developed based on ultra-high-performance liquid chromatography coupled with triple quadrupole mass spectrometry. Analysis was performed on an Agilent 1290 Infinity II series UHPLC system, equipped with a Waters ACQUITY UPLC HSS T3 column (100 × 2.1 mm, 1.8 *μ*m) by using (A) 0.1% acetic acid and (B) methanol as mobile phase. The flow rate was 0.3 mL/min, and the injection volume was 1 *μ*L. Mass spectrometry was operated in multiple reaction monitoring mode using an Agilent 6460 triple quadrupole mass spectrometer equipped with an AJS-ESI ion source. Agilent Mass Hunter Work Station Software was employed for data acquisition and processing. All calibration curves showed excellent linear regressions (*R*^2^ > 0.9992). The precision, repeatability, and stability of the ten compounds were below 4.56% in terms of relative standard deviation. The average extraction recovery ranged from 96.53% to 102.69% with a relative standard deviation of 1.14–3.78% for all samples. This study potently contributes to the quantitative evaluation of Ge-Gen-Jiao-Tai-Wan, thereby providing a scientific basis for further studies and clinical application of Ge-Gen-Jiao-Tai-Wan.

## 1. Introduction

Traditional Chinese medicine (TCM) is gaining attention from the international community because of the holistic concept of the TCM theory and historical clinical practice. TCM, not only acts as an extremely important part of China's medical and health causes, but is also a shining pearl in the treasure house of human traditional medicine culture. As China's unique medical characteristics, TCM owns rich clinical experience and huge potential therapeutic value in the long-term practice process. The Ge-Gen-Jiao-Tai-Wan (GGJTW) formula, derived from the prior and well-known TCM formula designated Jiao-Tai-Wan, is composed of Kudzu root (Ge-Gen in Chinese), Rhizoma coptidis (Huang-Lian in Chinese), and Cinnamon (Rou-Gui in Chinese), and it has been a classic formula for the treatment of type 2 diabetes mellitus (T2DM) in Central South University, Xiangya Hospital for many years. Our previous studies have proved that GGJTW contributes greatly to the amelioration of hyperglycaemia in T2DM [1, 2]. Kudzu root, Rhizoma coptidis, and cinnamon, first recorded in the ancient TCM book “Shen-Nong-Ben-Cao-Jing” (Han Dynasty), are nowadays famous Chinese herbs documented in the Pharmacopoeia of the People's Republic of China (2015 edition).

Modern pharmacological research studies have revealed some important compounds such as puerarin, daidzin, and daidzein in Kudzu root; berberine, palmatine, coptisine, epiberberine, and jatrorrhizine in Rhizoma coptidis; cinnamic acid and coumarin mainly exist as volatile oil in cinnamon, which are found to be responsible for the biological activities in the three single herbs and proved to be the active components [3–5]. Various work published in the literature have established that these compounds possess extensive biomedical effects on anti-inflammatory [6–8], antidiabetes [9–11], hypolipidemic effects [12–14], antitumor activity [15–17], and improving cardiocerebrovascular functions [18–20].

The chemical components contained in TCM compounds are complex with uneven content, and those effective components are the material basis of the biological effect of the whole formula. Through the simultaneous qualitative and quantitative analysis, the content of various chemical components and the proportion of each component can be determined, hence realizing the quality control of TCM compounds [21–26]. However, to the best of our knowledge, the methods for determining components used in previous reports only focused on one or two herbs of the whole preparation of GGJTW [1, 27–29]. Fang et al. developed a method based on the high-performance capillary electrophoresis (HPCE) method with diode array detection (DAD) for the separation and determination of isoflavonoids in Kudzu root [27]. Kong et al. simultaneously quantified five active alkaloids and chemical fingerprint analysis for quality control of Rhizoma coptidis chinensis based on UPLC-PAD combined with chemometrics methods [28]. Foudah et al. reported the determination of cinnamaldehyde and eugenol in cinnamon using a sustainable/green HPTLC technique [29]. In our previous study, we only determined the content of puerarin and berberine in the respective herbs and in the final GGJTW extracts by UPLC-PAD, lacking the identification of multiple compounds in the whole formula [1]. As previously mentioned, there have been no reports specifically focusing on quantitative determination of the whole GGJTW formula. Therefore, a rapid, accurate, and sensitive method is emergent to quantify the compounds in GGJTW, which is conducive for the quality control, searching the multiple active compositions of the classical TCM formula, and illustrating the deeper meaning of combined use of single herbs. Nowadays, ultrahigh-performance liquid chromatography coupled with triple quadrupole mass spectrometry (UHPLC-QQQ-MS) has become a pivotal and potent instrument in analyzing substances with a lower content of complicated TCM formulas because of its high resolution and outstanding sensitivity [30–32]. In this regard, our present study conducted a systematic and comprehensive analysis by UHPLC-QQQ-MS to fully illuminate the chemical constituents of GGJTW for the first time.

Herein, we successfully established a UHPLC-QQQ-MS analytical method for simultaneous determining ten active compounds (puerarin, daidzin, coptisine, epiberberine, jatrorrhizine, berberine, palmatine, coumarin, daidzein, and cinnamic acid) in GGJTW, and the method was well validated for quality evaluation based on specificity, linearity, precision, recovery, repeatability, and stability. The proposed methodology of quantitative determination contributed greatly for the detection of the ten targeted compounds in the GGJTW extract and can be used for further quality control, thereby establishing a forceful basis for the further study of its efficacy and safety in clinical applications.

## 2. Materials and Methods

### 2.1. Chemicals and Reagents

**Figure 1:**
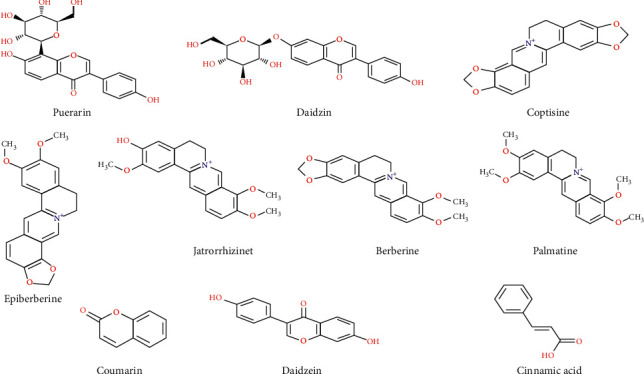
Chemical structures of 10 standard reference compounds in GGJTW.

Standards including puerarin, daidzin, coptisine, epiberberine, jatrorrhizine, berberine, palmatine, coumarin, daidzein, and cinnamic acid (purity >98%) were purchased from Chengdu Pufei De Biotech Co., Ltd. (Chengdu, China). The chemical structures of the abovementioned ten components are shown in Figure 1, respectively. Acetic acid and methanol (LC-MS grade) were obtained from Sigma-Aldrich (St. Louis, MO, USA) Inc. (CAS: 64-19-7; batch number: A6283) and ANPEL Laboratory Technologies (Shanghai) Inc. (CAS: 67-56-1; batch number: F2690144), respectively. Deionized water was prepared by the Milli-Q system (Millipore, Billerica, MA, USA).

### 2.2. Preparation of GGJTW Extracts

The extraction of GGJTW was based on our previous literature report [1] and strictly followed the drug production standard. The extraction was approved by professor Yanmei Peng, Institute of Traditional Chinese Medicine, Hunan University of Chinese Medicine (Hunan, China) and was completely implemented in Hunan Guo-Hua Pharmaceuticals Ltd. (Hunan, China). The main ingredient of *cinnamon* is volatile oil, thus the extraction procedure of the formula is to extract cinnamon oil first and then mix the liquid for further water-extraction [33]. The final calculated extract yield of GGJTW was 18.37%, and the content of volatile oil was 1.38% (v/w, mL/g) in *cinnamon*, the total puerarin content in *Kudzu root* was 2.5% (w/w), and the total berberine content in *Rhizoma coptidis* was 5.8% (w/w). Thus, these major components were compliant with the herb quality standards of the Chinese Pharmacopoeia (2015 edition). The GGJTW extracts were stored in a 4°C refrigerator for use.

### 2.3. Chromatographic Conditions

An ultrahigh-performance liquid chromatograph was implemented on a 1290 Infinity II series UHPLC System (Agilent Technologies). The analytical column was a Waters ACQUITY HSS T3 column (100 × 2.1 mm, 1.8 *μ*m) from Waters Co. (USA) with a temperature of 40°C. (A) 0.1% acetic acid aqueous solution and (B) methanol constituted the two parts of the mobile phase at the following gradient elution procedures (0–1 min, 70–70% A; 1–6 min, 70–5% A; 6–10 min, 5–5% A; 10–10.5 min, 5–70% A; 10.5–14 min, 70–70% A). The flow rate was controlled at 0.3 mL/min, and the temperature of the sample tray was set at 4°C. The injection volume was 1 *μ*L.

### 2.4. Mass Spectrometry Conditions

An Agilent 6460 triple quadrupole mass spectrometer (Agilent Technologies), equipped with an AJS electrospray ionization (AJS-ESI) interface, was applied to perform mass spectrometry in multiple reaction monitoring (MRM) mode. Typical ion source parameters were as follows: capillary voltage = +4000/-3500 V, nozzle voltage = +500/-500 V, atomizing gas (N2) temperature = 300°C, atomizing gas (N2) flow rate = 5 L/min, sheath gas (N2) temperature = 250°C, sheath gas (N2) flow rate = 11 L/min, and nebulizer = 45 psi. The MRM parameters for each of the targeted analytes were optimized using flow injection analysis by injecting the standard solutions of the individual analytes into the API source of the mass spectrometer. Several most sensitive transitions were used in the MRM scan mode to optimize the collision energy for each Q1/Q3 pair (Table 1). Among the optimized MRM transitions per analyte, the Q1/Q3 pairs that showed the highest sensitivity and selectivity were selected as “quantifier” for quantitative monitoring. The additional transitions acted as “qualifier” for the purpose of verifying the identity of the target analytes. Agilent MassHunter Work Station Software (B.08.00, Agilent Technologies) was employed for MRM data acquisition and processing [34].

### 2.5. Preparation of Standard and Quality Control (QC) Solutions

Puerarin 4.096 mg, daidzin 3.836 mg, coptisine 1.788 mg, epiberberine 0.942 mg, jatrorrhizine 1.812 mg, berberine 0.955 mg, palmatine 0.974 mg, coumarin 1.468 mg, daidzein 0.611 mg, and 7.390 mg of cinnamic acid were accurately weighed and were separately placed in a 10 mL brown volumetric flask. Methanol was added and then dissolved by ultrasound to obtain the reserve solution of ten reference substances. The concentrations were 409.6, 383.6, 178.8, 94.2, 181.2, 95.5, 97.4, 146.8, 61.1, and 739.0 *μ*g/mL, respectively. Another 10 mL brown flask was taken, and 5 mL of methanol and 50 *μ*L of each of the ten reference substances reserve solutions were added, respectively, and diluted to 10 mL with methanol. The concentration of each analyte in standard mixture solutions was as follows: 2.0480 *μ*g/mL for puerarin, 1.9180 *μ*g/mL for daidzin, 0.8940 *μ*g/mL for coptisine, 0.4710 *μ*g/mL for epiberberine, 0.9060 *μ*g/mL for jatrorrhizine, 0.4775 *μ*g/mL for berberine, 0.4870 *μ*g/mL for palmatine, 0.7340 *μ*g/mL for coumarin, 0.3055 *μ*g/mL for daidzein, and 3.6950 *μ*g/mL for cinnamic acid. Then, the standard mixture solution was successively diluted by 2 times to prepare a series of standard solutions containing 9 concentration points to establish calibration curves. QC samples were prepared at three concentration levels containing puerarin (0.1307, 0.5440, and 2.0480 *μ*g/mL), daidzin (0.1440, 0.5753, and 1.9180 *μ*g/mL), coptisine (0.0514, 0.2231, and 0.8940 *μ*g/mL), epiberberine (0.0261, 0.1136, and 0.4710 *μ*g/mL), jatrorrhizine (0.0583, 0.2424, and 0.9060 *μ*g/mL), berberine (0.0259, 0.1123, and 0.4775 *μ*g/mL), palmatine (0.0286, 0.1220, and 0.4870 *μ*g/mL), coumarin (0.0464, 0.1834, and 0.7340 *μ*g/mL), daidzein (0.0197, 0.0833, and 0.3055 *μ*g/mL), and cinnamic acid (0.2153, 0.9600, and 3.6950 *μ*g/mL).

### 2.6. Preparation of GGJTW Solution

1.2 g of GGJTW extract (crude herb, 6.53 g) was accurately weighed and added to 40 mL of deionized water, followed by ultrasound for 10 min to a concentration of 30 mg/mL of GGJTW solution. A 10 *μ*L aliquot of the sample was precisely transferred to an Eppendorf tube. After the addition of 990 *μ*L of 75% methanol, the samples were vortexed for 30 s, and centrifuged at 12000 rpm (4°C) for 15 min. Finally, a 60 *μ*L aliquot of the clear supernatant was transferred to an auto-sampler vial and 1 *μ*L of volume was sucked into the system for UHPLC-QQQ-MS analysis (dilution factor = 100). Thus, the sample was diluted by 100 times for the determination of coumarin, daidzein, and cinnamic acid. In addition, the supernatant was further diluted 10 times with 75% methanol for the determination of puerarin, daidzin, coptisine, epiberberine, jatrorrhizine, berberine, and palmatine (dilution factor = 1000).

## 3. Results and Discussion

### 3.1. Optimization of the UHPLC Chromatographic Conditions

**Figure 2:**
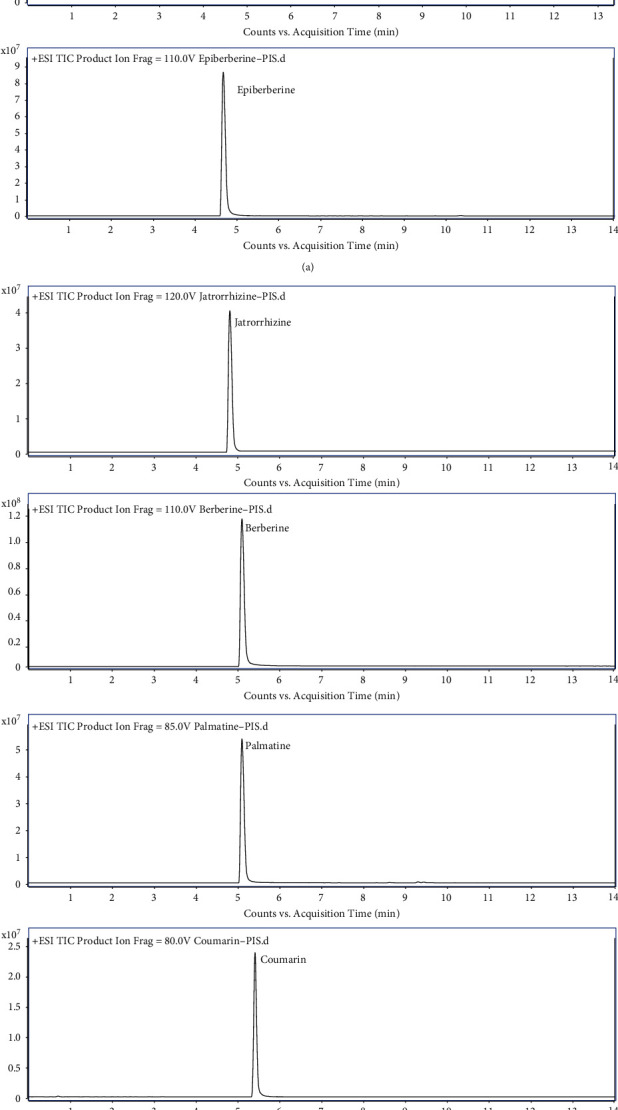
UHPLC-QQQ-MS/MS extracted ion chromatograms of 10 investigated compounds.

The UHPLC conditions were principally determined by selecting columns and optimizing the compositions of the mobile phase and gradient elution programs for the rapid and effective separation of the chemical constituents in GGJTW. It was tested that the Waters ACQUITY HSS T3 column (100 × 2.1 mm, 1.8 *μ*m) from Waters Co. (USA) achieved high column efficiency and excellent separation of multiple compounds in this study. Moreover, in our preliminary test, we investigated the mobile phase systems of acetonitrile-water and methanol-water. It was found that methanol-water could well separate the chromatographic peaks in the GGJTW prescription, and the peak capacity was large. Phosphoric acid and acetic acid were also added to the mobile phase for adjusting the pH or polarity. A good peak shape can be obtained when the concentration of acetic acid was 0.1%, and the measured components can be separated from the interference peak to the baseline, meanwhile, most of the components had good response by mass spectrum. As a result, 0.1% acetic acid aqueous solution and methanol were chosen as the preferred mobile phases. Furthermore, because of the complexity of the compounds and the significant differences between respective contents, gradient elution was used to effectively separate the compounds to the greatest extent. By optimizing the gradient elution conditions, the UHPLC system employed a gradient elution of 0.1% acetic acid aqueous solution (A) and methanol (B) (0–1 min, 70–70% A; 1–6 min, 70–5% A; 6–10 min, 5–5% A; 10–10.5 min, 5–70% A; 10.5–14 min, 70–70% A) with a 1 *μ*L injection volume at a mobile phase flow rate of 0.3 mL/min, obtaining good resolution and peak shape. And the temperature of the sample tray was set at 4°C, the column temperature was kept at 40°C. The typical extracted ion chromatograms (EIC) of the ten components are presented in Figure 2.

### 3.2. Optimization of MS Conditions

**Figure 3:**
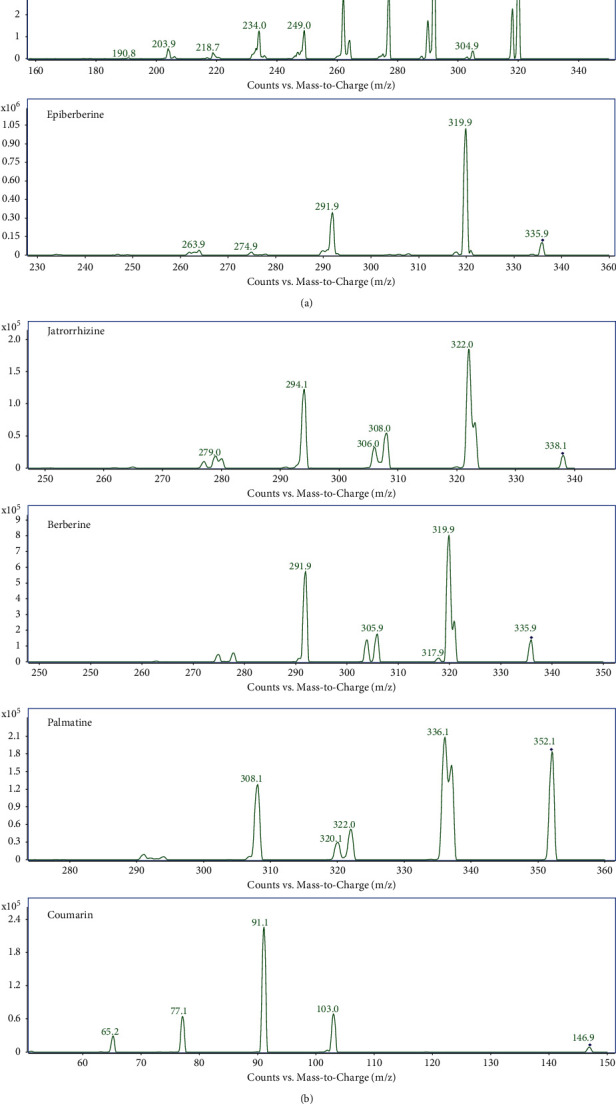
Product ion mass spectra of [M + H]+ ions of puerarin, daidzin, coptisine, epiberberine, jatrorrhizine, berberine, palmatine, coumarin, and daidzein; [M-H]- ions of cinnamic acid.

Both positive and negative ion scanning modes were implemented for qualitative analysis of the compounds due to the variety of chemical constituents in the extracts and their different response modes. By adjusting the fragment voltage of the mass spectrum, we found that most of the excimer ion peaks of the chemical components are stable when the fragment voltage is between 80–120 V, and a certain amount of fragments will be generated, which is conducive to the qualitative analysis of the compounds. Several parent-daughter ion pairs (transitions) with the highest signal intensity were selected to optimize the MRM parameters, with atomizer pressure determined to be 45 psi, atomizing gas flow rate to be 5 L/min, and sheath flow rate at 11 L/min. The capillary voltage is 4000 V for the positive ion mode and 3500 V for the negative ion mode as conventional value. Mass spectrometry results showed that cinnamic acid only responded in the negative ion mode, and the other nine compounds responded well in the positive ion mode. Product ion mass spectra of [M + H]+ ions of puerarin, daidzin, coptisine, epiberberine, jatrorrhizine, berberine, palmatine, coumarin, and daidzein; [M-H]- ions of cinnamic acid in the GGJTW extract are shown in Figure 3.

### 3.3. Method Validation

The developed method was assessed in terms of specificity, linearity, LOD, LOQ, precision, accuracy, stability, and repeatability, and followed with Guidance for Industry-Bioanalytical Method Validation by the U.S. Food and Drug Administration (FDA).

#### 3.3.1. Specificity

**Figure 4:**
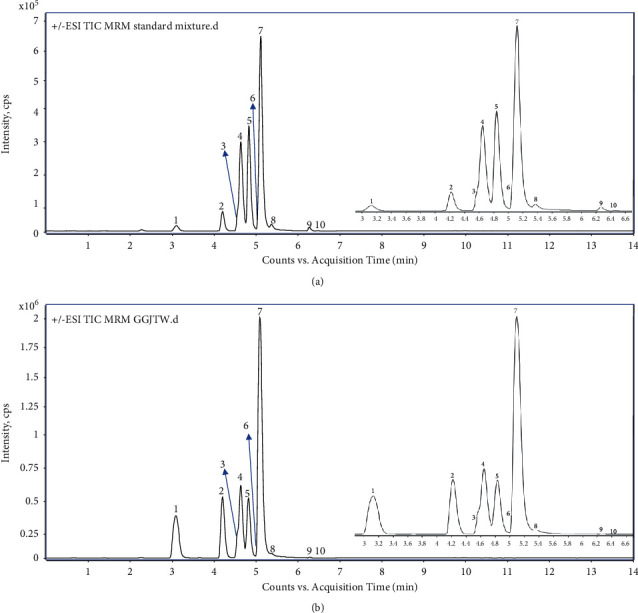
Representative MRM chromatograms of (a) standard mixture and (b) GGJTW extract: puerarin (1), daidzin (2), coptisine (3), epiberberine (4), jatrorrhizine (5), berberine (6), palmatine (7), coumarin (8), daidzein (9), and cinnamic acid (10).

The specificity test was performed by evaluating the overlaid chromatograms between the standard solutions and GGJTW sample solutions for significant interfering peaks at the retention times of puerarin (1), daidzin (2), coptisine (3), epiberberine (4), jatrorrhizine (5), berberine (6), palmatine (7), coumarin (8), daidzein (9), and cinnamic acid (10) peaks. The chromatograms of the standard mixture and GGJTW with good separation are illustrated in Figure 4, with retention times of 1–10 at 3.12, 4.22, 4.58, 4.65, 4.83, 5.08, 5.14, 5.39, 6.27, and 6.46 min, respectively. There were no significant differences in retention time and chromatographic peak shape between the biological sample and the standard solution.

#### 3.3.2. Linearity

Taking the measured standard peak area of each component *y* as the ordinate and the concentration of the target compound *x* as the abscissa, linear regression was carried out by using the least square method, and the weight was set as 1/*x* to obtain the linear range and regression equation of each component. As illustrated in Table 2, linear regression analysis of the 10 components in the respective concentration range showed good linearity for each analyte (*R*^2^ > 0.9992), which allowed for the acquisition of reliable and effective data for the analyzed samples.

#### 3.3.3. LOD and LOQ

The limit of detection (LOD) and limit of quantification (LOQ) were determined at a signal-to-noise ratio (S/N) of 3 and 10, respectively. The LODs were 1.12, 0.88, 0.75, 0.25, 0.5, 0.31, 0.28, 0.43, 0.17, and 2.09 ng/mL for puerarin, daidzin, coptisine, epiberberine, jatrorrhizine, berberine, palmatine, coumarin, daidzein, and cinnamic acid, respectively. The LOQs were 3.93, 3.01, 2.32, 0.86, 1.73, 1.15, 0.90, 1.44, 0.63, and 6.46 ng/mL for puerarin, daidzin, coptisine, epiberberine, jatrorrhizine, berberine, palmatine, coumarin, daidzein, and cinnamic acid, respectively.

#### 3.3.4. Precision

The intra and interday variations were for determining the precision of the developed method. Relative standard deviation (RSD) was utilized as a measurement of precision. Intra and interday precision were determined on 6 replicates of QC samples at 3 different concentrations within 1 day or 3 consecutive days, respectively. The peak area of each component was recorded for UHPLC-QQQ-MS analysis, and the regression equation was employed to calculate the concentration of 10 components in each injection. Results concerning the precision of this developed method are exhibited in Table S1 (Supplementary Material). Overall, the RSD of intraday precision ranged from 1.02% to 3.66%, and interday precision ranged from 1.14% to 3.39%.

#### 3.3.5. Accuracy

The accuracy of the analytical method was evaluated by using the recovery test. Recoveries of 10 compounds on six replicates were investigated by spiking with the authentic standards to the GGJTW samples. Peak areas of each analyte in six GGJTW samples at three different concentration levels (80%, 100%, and 120% of the known amounts) were recorded. Then, the concentrations of the 10 compounds in the GGJTW samples after spiking were calculated according to the peak area using the calibration curve. The average recovery percentage was calculated by the following formula: recovery (%) = (total amount after spiking−original amount in sample)/spiked amount × 100%. The average extraction recovery ranged from 96.53% to 102.69%, and the RSDs were below 3.78% (Table S2, Supplementary Material), indicating that the method could ensure the acquisition of accurate and consistent data for all the constituents.

#### 3.3.6. Stability

The stability of the analytical GGJTW solution at environmental temperature was studied by detecting the sample solution at 0, 2, 4, 8, 12, and 24 h. The RSD values of peak areas were taken for assessment.  Table S3 (Supplementary Material) shows the stability tests. The RSD values of the 10 compounds were below 4.56%. The data confirmed that there was no significant degradation and all the 10 compounds in the GGJTW solution had good stability within 0–24 h.

#### 3.3.7. Repeatability

In order to verify the repeatability of the method, six different sample solutions prepared from the same GGJTW sample were analyzed in parallel by the method described in Section 2.3–2.4.  Table S4 (Supplementary Material) exhibited that the RSD values of the ten chemical constituents were below 4.44%, which confirmed the high reproducibility of the method.

### 3.4. Quantitative Determination of 10 Compounds in GGJTW Extract

This newly developed analytical method was subsequently applied to determine the 10 constituents in the GGJTW extract. Three replicates of GGJTW extract (1.2 g) were accurately weighed, and the sample solution was prepared according to the method in Section 2.6. 1 *μ*L of GGJTW solution was sucked into the system for quantitative analysis, and the peak area was counted to calculate the contents of the 10 components in the GGJTW extract. Typical MRM chromatograms of the standard mixture and GGJTW extract are shown in Figure 4. The retention time and peak shapes for all of the analytes showed good correlation between the standard solution and the real sample.  Table 3 demonstrates that puerarin, daidzin, coptisine, epiberberine, jatrorrhizine, berberine, palmatine, coumarin, daidzein, and cinnamic acid in GGJTW were detected successfully. The contents in the GGJTW extract were puerarin (57.32 ± 1.21 mg/g), daidzin (21.37 ± 0.66 mg/g), coptisine (3.42 ± 0.14 mg/g), epiberberine (2.56 ± 0.09 mg/g), jatrorrhizine (2.08 ± 0.04 mg/g), berberine (10.93 ± 0.41 mg/g), palmatine (3.22 ± 0.10 mg/g), coumarin (0.46 ± 0.01 mg/g), daidzein (0.40 ± 0.01 mg/g), and cinnamic acid (0.50 ± 0.02 mg/g), respectively, and the overall RSD was below 4.10%. There were significant differences in the content of the 10 components. Puerarin is the most abundant constituent, accounting for 5.73% of the total content. Daidzin and berberine followed with 2.14% and 1.09%, respectively. The proportions of coptisine, epiberberine, jatrorrhizine, and palmatine were relative closer, significantly lower than those of puerarin, daidzin, and berberine. The content of coumarin, daidzein, and cinnamic acid was similar at a lower level, and daidzein was the lowest, only accounting for 0.04% of the total GGJTW content.

## 4. Conclusions

This work demonstrated a detailed research for quantitative determination in GGJTW. To the best of our knowledge, this is the first report using the UHPLC-QQQ-MS method for simultaneously qualitative and quantitative determination of puerarin, daidzin, coptisine, epiberberine, jatrorrhizine, berberine, palmatine, coumarin, daidzein, and cinnamic acid in GGJTW extract. The established method could well identify the contents of the abovementioned ten components. In terms of methodology, the standard curves of the ten compounds were successfully established, and the linearity, precision, recovery rate, stability, and reproducibility of the method were fully validated. This effective, sensitive, rugged, and safe method can be used for further quality control of GGJTW. Our systematic study of the chemical constituents of GGJTW extract by the developed UHPLC-QQQ-MS method provides a basis for further scientific studies on GGJTW and a foundation for the effective development and application of GGJTW.

## Figures and Tables

**Table 1:** Analytical MRM parameters of the developed UHPLC-QQQ-MS method.

Analyte	Precursor ion	Product ion	Fragmentor (V)	CE (V)	Polarity	Quantifier/qualifier
Puerarin	417.1	297.1	90	32	Positive	Quantifier
	417.1	267.0	90	32	Positive	Qulifier

Daidzin	417.1	255.0	90	15	Positive	Quantifier
	417.1	199.0	90	50	Positive	Qulifier

Coptisine	320.1	292.0	110	30	Positive	Quantifier
	320.1	277.0	110	40	Positive	Qulifier

Epiberberine	335.9	319.9	110	32	Positive	Quantifier
	335.9	291.9	110	35	Positive	Qulifier

Jatrorrhizine	338.1	322.0	120	32	Positive	Quantifier
	338.1	294.1	120	28	Positive	Qulifier

Berberine	335.9	319.9	110	32	Positive	Quantifier
	335.9	291.9	110	35	Positive	Qulifier

Palmatine	352.1	336.1	85	30	Positive	Quantifier
	352.1	308.1	85	28	Positive	Qulifier

Coumarin	146.9	91.1	80	25	Positive	Quantifier
	146.9	103.0	80	15	Positive	Qulifier

Daidzein	254.9	91.1	100	40	Positive	Quantifier
	254.9	199.0	100	25	Positive	Qulifier

Cinnamic acid	146.8	103.0	80	7	Negative	Quantifier

**Table 2:** Regression equation, correlation coefficient, linear range, LOD, and LOQ of 10 components in UHPLC-QQQ-MS.

Analyte	Regression equation	Correlation Coefficient (R2)	Linear range (*µ*g/mL)	LOD (ng/mL)	LOQ (ng/mL)
Puerarin	*y* = 181790.01 *x* + 29.65	0.9995	0.00774～2.048	1.12	3.93
Daidzin	*y* = 703318.66 *x* + 481.60	0.9992	0.00826～1.918	0.88	3.01
Coptisine	*y* = 926264.11 *x* *–* 1293.07	0.9998	0.00444～0.894	0.75	2.32
Epiberberine	*y* = 2870685.59 *x* + 965.63	0.9996	0.00174～0.471	0.25	0.86
Jatrorrhizine	*y* = 2719691.61 *x* *–* 352.51	0.9995	0.00323～0.906	0.50	1.73
Berberine	*y* *=* 2845616.29*x* – 1165.64	0.9994	0.00175～0.4775	0.31	1.15
Palmatine	*y* = 3200432.28 *x* *–* 521.16	0.9994	0.00175～0.487	0.28	0.90
Coumarin	*y* = 251481.47 *x* *–* 53.44	0.9998	0.00283～0.734	0.43	1.44
Daidzein	*y* = 120736.64 *x* + 16.89	0.9995	0.00112～0.3055	0.17	0.63
Cinnamic acid	*y* = 6148.94 *x* *–* 6.12	0.9996	0.01633～3.695	2.09	6.46

*y*: peak area of the components; *x*: concentration in *µ*g/mL.

**Table 3:** Content determination of 10 components in GGJTW (n = 3).

Components	Contents (mg/g）			Mean ± SD （mg/g）	RSD （%）	Ratio （%）
	GGJTW 1	GGJTW 2	GGJTW 3			
Puerarin	58.39	57.58	56.00	57.32 ± 1.21	2.12	5.73
Daidzin	21.80	21.70	20.60	21.37 ± 0.66	3.11	2.14
Coptisine	3.53	3.46	3.26	3.42 ± 0.14	4.10	0.34
Epiberberine	2.65	2.57	2.46	2.56 ± 0.09	3.69	0.26
Jatrorrhizine	2.12	2.09	2.04	2.08 ± 0.04	1.76	0.21
Berberine	11.17	11.16	10.46	10.93 ± 0.41	3.75	1.09
Palmatine	3.29	3.26	3.10	3.22 ± 0.10	3.18	0.32
Coumarin	0.47	0.46	0.45	0.46 ± 0.01	2.36	0.046
Daidzein	0.41	0.40	0.40	0.40 ± 0.01	1.86	0.04
Cinnamic acid	0.51	0.50	0.48	0.50 ± 0.02	3.85	0.05

## Data Availability

The data used to support the findings of this study are included within the article, and any further information is available from the corresponding author upon request.

## Supplementary Materials

Supplementary Materials The method validation in terms of precision, accuracy, stability, and repeatability for GGJTW determined by UHPLC-QQQ-MS is detailed in the supplementary materials. Table S1: precision of ten components in UHPLC-QQQ-MS. Table S2: spike recoveries of ten components in GGJTW. Table S3: stability of ten components in GGJTW. Table S4: repeatability of ten components in GGJTW.
