# Supplementary material for: Development and Validation of Ultrahigh-Performance Liquid Chromatography Coupled with Triple Quadrupole Mass Spectrometry Method for Quantitative Determination of Ten Active Compounds in Ge-Gen-Jiao-Tai-Wan
Source: J Anal Methods Chem. 2022 Apr 10;2022:4713799. doi: 10.1155/2022/4713799 (PMC9013549; doi:10.1155/2022/4713799)
Supplement: Supplementary Materials — The method validation in terms of precision, accuracy, stability, and repeatability for GGJTW determined by UHPLC-QQQ-MS is detailed in the supplementary materials. Table S1: precision of ten components in UHPLC-QQQ-MS. Table S2: spike recoveries of ten components in GGJTW. Table S3: stability of ten components in GGJTW. Table S4: repeatability of ten components in GGJTW. [file 4713799.f1.doc]

**Journal of Analytical Methods in Chemistry**

**Supplementary Material**

**Development and Validation of Ultra-High-Performance Liquid Chromatography Coupled with Triple Quadrupole Mass Spectrometry Method for Quantitative Determination of Ten Active Compounds in Ge-Gen-Jiao-Tai-Wan**

Wenbo Wang^1, 2^, Shuangquan Zhu^3^, Hao Chen^4^, Ning Wu^5^, Han Chen^1, 2^, Dongsheng Wang^1, 2, *^

*^1^ Department of Integrated Traditional Chinese and Western Medicine, Xiangya Hospital, Central South University, Changsha, 410008, China.*

*^2^* *National Clinical Research Center for Geriatric Diseases, Xiangya Hospital, Central South University, Changsha, 410008, China.*

*^3^ Department of gynecology, The Second Affiliated Hospital of Hunan University of Chinese Medicine, Changsha, 410005, China.*

*^4^ Department of clinical laboratory, The First Affiliated Hospital of Hunan University of Chinese Medicine, Changsha, 410007, China.*

*^5^ Changsha Social Work College, Changsha, 410116, China.*

Correspondence should be addressed to Dongsheng Wang; wdsh666@126.com

Table S1: Precision of ten components in UHPLC-QQQ-MS (n=6).

| Analyte | Spiked conc.  (µg/mL) | Intra-day precision | | Inter-day precision | |
| --- | --- | --- | --- | --- | --- |
|  |  | Mean ± SD  (µg/mL) | RSD  (%) | Mean ± SD  (µg/mL) | RSD  (%) |
| Puerarin | 0.1307 | 0.1308±0.0030 | 2.29 | 0.1303±0.0017 | 1.32 |
|  | 0.5440 | 0.5423±0.0141 | 2.60 | 0.5362±0.0111 | 2.08 |
|  | 2.0480 | 2.0527±0.0332 | 1.62 | 2.0510±0.0362 | 1.77 |
| Daidzin | 0.1440 | 0.1438±0.0046 | 3.23 | 0.1427±0.0034 | 2.41 |
|  | 0.5753 | 0.5701±0.0126 | 2.20 | 0.5676±0.0177 | 3.11 |
|  | 1.9180 | 1.9371±0.0251 | 1.29 | 1.9544±0.0563 | 2.88 |
| Coptisine | 0.0514 | 0.0521±0.0011 | 2.12 | 0.0513±0.0013 | 2.60 |
|  | 0.2231 | 0.2238±0.0040 | 1.80 | 0.2216±0.0054 | 2.42 |
|  | 0.8940 | 0.8977±0.0194 | 2.16 | 0.8962±0.0130 | 1.45 |
| Epiberberine | 0.0261 | 0.0259±0.0006 | 2.36 | 0.0266±0.0007 | 2.63 |
|  | 0.1136 | 0.1131±0.0030 | 2.67 | 0.1137±0.0013 | 1.16 |
|  | 0.4710 | 0.4698±0.0063 | 1.34 | 0.4709±0.0071 | 1.51 |
| Jatrorrhizine | 0.0583 | 0.0586±0.0011 | 1.82 | 0.0589±0.0009 | 1.48 |
|  | 0.2424 | 0.2418±0.0025 | 1.02 | 0.2428±0.0045 | 1.84 |
|  | 0.9060 | 0.9066±0.0110 | 1.21 | 0.9059±0.0103 | 1.14 |
| Berberine | 0.0259 | 0.0261±0.0008 | 3.18 | 0.0262±0.0006 | 2.45 |
|  | 0.1123 | 0.1131±0.0022 | 1.94 | 0.1121±0.0017 | 1.56 |
|  | 0.4775 | 0.4753±0.0056 | 1.17 | 0.4751±0.0161 | 3.39 |
| Palmatine | 0.0286 | 0.0289±0.0007 | 2.44 | 0.0289±0.0006 | 2.08 |
|  | 0.1220 | 0.1226±0.0019 | 1.58 | 0.1232±0.0024 | 1.93 |
|  | 0.4870 | 0.4808±0.0139 | 2.90 | 0.4852±0.0068 | 1.41 |
| Coumarin | 0.0464 | 0.0466±0.0013 | 2.87 | 0.0463±0.0013 | 2.75 |
|  | 0.1834 | 0.1840±0.0033 | 1.77 | 0.1820±0.0028 | 1.54 |
|  | 0.7340 | 0.7358±0.0147 | 1.99 | 0.7373±0.0212 | 2.87 |
| Daidzein | 0.0197 | 0.0195±0.0003 | 1.78 | 0.0197±0.0005 | 2.63 |
|  | 0.0833 | 0.0838±0.0031 | 3.66 | 0.0831±0.0017 | 2.10 |
|  | 0.3055 | 0.3073±0.0087 | 2.83 | 0.3047±0.0069 | 2.28 |
| Cinnamic acid | 0.2153 | 0.2165±0.0025 | 1.16 | 0.2143±0.0043 | 2.03 |
|  | 0.9600 | 0.9641±0.0118 | 1.22 | 0.9614±0.0144 | 1.50 |
|  | 3.6950 | 3.6819±0.0840 | 2.28 | 3.6695±0.0654 | 1.78 |

Table S2: Spike recoveries of ten components in GGJTW (n=6).

| Analyte | Original  (µg) | Spiked  (µg) | Measured  (µg) | Measured content(µg)  Mean ± SD | Averaged Recovery  (%) | RSD  (%) |
| --- | --- | --- | --- | --- | --- | --- |
| Puerarin | 1.3916 | 1.3916 | 2.7834 | 2.7653±0.0177 | 98.71 | 1.29 |
|  | 1.7395 | 1.7395 | 3.4755 | 3.4746±0.0277 | 99.75 | 1.60 |
|  | 2.0874 | 2.0874 | 4.1751 | 4.1841±0.0245 | 100.44 | 1.17 |
| Daidzin | 0.5086 | 0.5086 | 1.0172 | 1.0153±0.0114 | 99.62 | 2.25 |
|  | 0.6358 | 0.6358 | 1.2708 | 1.2834±0.0185 | 101.85 | 2.86 |
|  | 0.7630 | 0.7630 | 1.5251 | 1.5332±0.0135 | 100.94 | 1.75 |
| Coptisine | 0.0830 | 0.0830 | 0.1651 | 0.1665±0.0030 | 100.56 | 3.62 |
|  | 0.1037 | 0.1037 | 0.2074 | 0.2050±0.0020 | 97.72 | 1.98 |
|  | 0.1244 | 0.1244 | 0.2489 | 0.2514±0.0021 | 102.12 | 1.64 |
| Epiberberine | 0.0626 | 0.0621 | 0.1241 | 0.1241±0.0015 | 99.09 | 2.40 |
|  | 0.0783 | 0.0783 | 0.1561 | 0.1545±0.0029 | 97.30 | 3.78 |
|  | 0.0940 | 0.0940 | 0.1882 | 0.1902±0.0029 | 102.32 | 2.97 |
| Jatrorrhizine | 0.0496 | 0.0496 | 0.0995 | 0.0998±0.0007 | 101.14 | 1.36 |
|  | 0.0620 | 0.0620 | 0.1248 | 0.1257±0.0016 | 102.69 | 2.59 |
|  | 0.0744 | 0.0744 | 0.1482 | 0.1477±0.0021 | 98.45 | 2.87 |
| Berberine | 0.2639 | 0.2639 | 0.5269 | 0.5196±0.0075 | 96.90 | 2.94 |
|  | 0.3299 | 0.3290 | 0.6581 | 0.6538±0.0057 | 98.44 | 1.75 |
|  | 0.3959 | 0.3959 | 0.7928 | 0.7938±0.0049 | 100.51 | 1.23 |
| Palmatine | 0.0766 | 0.0766 | 0.1534 | 0.1536±0.0019 | 100.46 | 2.52 |
|  | 0.0958 | 0.0958 | 0.1918 | 0.1933±0.0031 | 101.79 | 3.17 |
|  | 0.1150 | 0.1150 | 0.2312 | 0.2307±0.0023 | 100.57 | 1.98 |
| Coumarin | 0.1095 | 0.1095 | 0.2188 | 0.2179±0.0024 | 99.00 | 2.24 |
|  | 0.1369 | 0.1369 | 0.2730 | 0.2748±0.0036 | 100.69 | 2.65 |
|  | 0.1643 | 0.1643 | 0.3286 | 0.3266±0.0037 | 98.79 | 2.25 |
| Daidzein | 0.1024 | 0.1021 | 0.2045 | 0.2051±0.0020 | 100.57 | 1.94 |
|  | 0.1280 | 0.1280 | 0.2545 | 0.2557±0.0026 | 99.77 | 2.04 |
|  | 0.1536 | 0.1536 | 0.3070 | 0.3043±0.0052 | 98.13 | 3.47 |
| Cinnamic acid | 0.1206 | 0.1206 | 0.2415 | 0.2402±0.0022 | 99.14 | 1.87 |
|  | 0.1507 | 0.1507 | 0.3008 | 0.2962±0.0045 | 96.53 | 3.09 |
|  | 0.1808 | 0.1808 | 0.3604 | 0.3611±0.0021 | 99.71 | 1.14 |

Table S3: Stability of ten components in GGJTW (n=6).

| Analyte | Content(ug/mL) | | | | | | |
| --- | --- | --- | --- | --- | --- | --- | --- |
|  | 0h | 2h | 4h | 8h | 12h | 24h | RSD (%) |
| Puerarin | 1.7516 | 1.7684 | 1.7204 | 1.7085 | 1.6907 | 1.7122 | 1.68 |
| Daidzin | 0.6541 | 0.6708 | 0.6892 | 0.6835 | 0.6709 | 0.6601 | 1.99 |
| Coptisine | 0.1058 | 0.1045 | 0.1023 | 0.1048 | 0.1099 | 0.1011 | 2.93 |
| Epiberberine | 0.0795 | 0.0851 | 0.0879 | 0.0827 | 0.0798 | 0.0782 | 4.56 |
| Jatrorrhizine | 0.0635 | 0.062 | 0.0651 | 0.0629 | 0.0616 | 0.0608 | 2.45 |
| Berberine | 0.3352 | 0.3408 | 0.3308 | 0.3265 | 0.3208 | 0.3316 | 2.09 |
| Palmatine | 0.0987 | 0.1001 | 0.1026 | 0.1009 | 0.0983 | 0.098 | 1.78 |
| Coumarin | 0.1397 | 0.1461 | 0.1435 | 0.1387 | 0.1451 | 0.1409 | 2.12 |
| Daidzein | 0.1231 | 0.1205 | 0.1207 | 0.1233 | 0.1254 | 0.1220 | 1.50 |
| Cinnamic acid | 0.1535 | 0.1546 | 0.1593 | 0.1534 | 0.1498 | 0.1524 | 2.04 |

Table S4: Repeatability of ten components in GGJTW (n=6).

| Analyte | 1 | 2 | 3 | 4 | 5 | 6 | Mean ± SD  （mg/g） | RSD  （%） |
| --- | --- | --- | --- | --- | --- | --- | --- | --- |
| Puerarin | 56.23 | 57.91 | 58.20 | 56.85 | 57.49 | 58.66 | 57.56±0.90 | 1.56 |
| Daidzin | 20.55 | 20.76 | 21.48 | 22.07 | 21.80 | 21.24 | 21.32 ±0.59 | 2.76 |
| Coptisine | 3.45 | 3.82 | 3.47 | 3.58 | 3.50 | 3.37 | 3.53± 0.16 | 4.44 |
| Epiberberine | 2.68 | 2.75 | 2.49 | 2.82 | 2.64 | 2.60 | 2.66± 0.12 | 4.34 |
| Jatrorrhizine | 2.08 | 2.11 | 2.14 | 2.06 | 2.09 | 2.07 | 2.09±0.03 | 1.40 |
| Berberine | 11.08 | 11.12 | 11.18 | 10.99 | 10.76 | 10.86 | 11.00± 0.16 | 1.47 |
| Palmatine | 3.12 | 3.28 | 3.23 | 3.19 | 3.20 | 3.16 | 3.20 ± 0.06 | 1.73 |
| Coumarin | 0.45 | 0.46 | 0.46 | 0.47 | 0.44 | 0.47 | 0.46± 0.01 | 2.55 |
| Daidzein | 0.40 | 0.40 | 0.41 | 0.41 | 0.41 | 0.42 | 0.41±0.01 | 1.84 |
| Cinnamic acid | 0.51 | 0.50 | 0.51 | 0.51 | 0.50 | 0.51 | 0.51± 0.01 | 1.02 |
